# Malingering in Adolescent Psychiatry: A Case Report of Fabricated Symptoms to Avoid Legal Consequences

**DOI:** 10.7759/cureus.60039

**Published:** 2024-05-10

**Authors:** Taylor F Faust, Jeremy W Shiver, Patrick G Dickinson, Elizabeth Vandervort, Maria Hamilton

**Affiliations:** 1 Department of Research, Alabama College of Osteopathic Medicine, Dothan, USA; 2 Department of Research, Edward Via College of Osteopathic Medicine, Auburn, USA; 3 Department of Child and Adolescent Psychiatry, BayPointe Hospital, Mobile, USA

**Keywords:** dsm 5, clinical psychology, pediatric neurology, pediatrics, malingering

## Abstract

Malingering is characterized by the deliberate fabrication and/or exaggeration of symptoms for secondary gain, posing a diagnostic challenge in healthcare settings. In this report, we present a 15-year-old male with a history of psychiatric disorders who attempted suicide to avoid legal sentencing, subsequently developing a stutter following an altercation with another patient. Despite initial concern for a concussion, further evaluation revealed malingering as the underlying motive. This case highlights the importance of identifying malingering in adolescents, which calls for a careful approach and thorough assessment for it to be distinguished from an authentic illness. Early identification of malingering optimizes resource allocation and ensures appropriate care for patients who have genuine medical needs.

## Introduction

Malingering is a falsification or exaggeration for secondary gain, including, but not limited to, seeking drugs, avoiding military service, or taking paid leave from a job [1]. The distinction between malingering and factitious disorder lies in the underlying motivations. Malingering typically stems from an external incentive or gain, while factitious disorder is driven by internal factors, such as the desire for recognition or validation. Identifying malingering can pose a challenge for healthcare professionals [2]. The training of healthcare workers emphasizes an inherent trust in patient report information, potentially leading to an underestimation of the prevalence of malingering in clinical encounters. Malingering and deception are prevalent in many social interactions today and are unlikely to be as rare as healthcare professionals expect [2]. For instance, some studies have shown that adolescents can effectively mimic symptoms of attention-deficit/hyperactivity disorder (ADHD) to secure prescriptions for stimulant medication [3]. This scenario is a common occurrence of malingering in an environment where adolescents are able to fake ADHD symptoms to acquire prescription stimulants. This behavior aims to enhance their academic performance or for recreational purposes, serving as two examples of secondary gain. In this report, we present the case of a 15-year-old male who attempted suicide by hanging and was admitted to an acute pediatric psychiatric hospital. He was later attacked by another patient and had a new-onset stutter.

## Case presentation

A 15-year-old male patient was admitted in 2024 to an acute pediatric psychiatric hospital after attempting suicide via hanging. He has had two prior psychiatric hospitalizations at the same hospital for poor impulse control in 2015 and 2019. His psychiatric history is significant for intermittent explosive disorder (IED), ADHD, oppositional defiant disorder (ODD), conduct disorder, and autism spectrum disorder without accompanying intellectual disability. His medications include serdexmethylphenidate and dexmethylphenidate (Azstarys), guanfacine (Intuniv), valproic acid (Depakote), and hydroxyzine (Vistaril).

His medical history includes one hospitalization for a hairline fracture in his right foot, and he denies any serious illnesses or major injuries. His social history consists of a 10th-grade education level. He lives at home with his father and younger sister. He has used cannabis in the past through smoking and consumption. There is no history of surgery. The patient is not allergic to any medicines.

On psychiatric evaluation, he had restricted affect, poor eye contact, and constant fidgeting of his hands. He was otherwise cooperative with the interview. He was admitted to inpatient hospitalization for a suicide attempt via hanging. He endorsed no suicidal ideation, homicidal ideation, or violent ideation and denied true intent to harm himself at this time. He reports remorse over the incident. He denies feeling depressed, changes in sleep, loss of interest, or changes in appetite. He denies symptoms of mania or hypomania.

On the physical exam, he is a well-developed, well-nourished, obese male with no lesions. When asked why he attempted suicide, he reported, “Because I wanted to avoid going to court.”

The hospital course was uneventful aside from one event. He complied with treatment plans, therapy sessions, and various group activities. He has not required sedating medications for aggressive behavior, nor has he made any suicidal gestures.

During admission, the patient was involved in an altercation with another patient and was bluntly attacked, being struck 18 times in the back of the head. Following the event, no immediate issues were reported by the staff. The following morning, upon interview, the patient demonstrated a newly acquired stutter, and he reported decreased visual acuity in his left eye, worsening nausea, one episode of emesis following the altercation, diffuse headache, posterior cervical muscle tenderness, and increased sensitivity to light and sounds. Nurses then examined the patient; they reported physical exam findings after the injury, including multiple scratches on the upper back and neck area with mild erythema. Cranial nerves I-XII were intact, with pupils equal bilaterally. The patient was sent to the hospital to get his head scanned, as he feared he had a serious head injury. The attending physician and nursing staff decided to send the patient to the emergency room, where he was evaluated for a head and neck injury. The CT scan of the head found no evidence of an acute intracranial process. The CT of the neck found no evidence of fractures. He was diagnosed with a closed head injury with a concussion.

The patient was interviewed again the following day. He featured continued stuttering and reported a continuing diffuse headache, worsening sensitivity to light and sounds, and nausea. His physical exam was unchanged from the previous day. The patient insisted that he had a “vacuum concussion,” as what they diagnosed him with at the emergency room, and that was causing his issues. The patient requested physical copies of his CT scans from the attending staff that day. The attending and nursing staff had an extensive conversation with the patient that evening. Due to the extensive time spent in the conversation, the patient’s stuttering started to become inconsistent, and a return to his normal speech became more apparent. The conversation ended with a return to the patient's baseline speech. On the third day following the event, the patient was interviewed, and his stuttering had resolved.

The patient revealed that after the incident, he created a stutter so that he would seem to have a major neurological injury to avoid his court sentencing. He was trying to utilize the hospital stay as a way to prolong and, if possible, avoid his return to the juvenile detention center and sentencing for his charges. Following this discussion, the patient had no more incidents and was discharged to a consequence-based program in stable condition.

The evidence presented in this patient's hospital placement and stay provides a narrative of the patient's behavior to avoid the charges placed against him. This behavior was confirmed by the patient himself after the attending and nursing staff became further involved in the patient interview following his emergency room visit. The patient's external goal is to put a false and profound exaggeration of stuttering to mimic a neurological injury to avoid his court ruling. In addition to his stuttering, his reasons for being placed at the psychiatric facility were on the terms of attempting to hang himself to avoid his trial date. The rest of his hospital stay featured increased anxiety and depressed mood due to his departure back to the juvenile detention center. His behavior featured two separate incidents of seeking secondary gain.

The patient's case was discussed with the attending physician and nursing staff, who took part in the care of the patient. With evidence of two separate events that sought to avoid the patient's upcoming trial, the patient was diagnosed with malingering.

## Discussion

A 15-year-old male with a history of autism spectrum disorder, conduct disorder, and multiple psychiatric hospitalizations presented to a child adolescent psychiatric facility after a suicide attempt coinciding with his legal sentencing. During evaluation, he denied depressive or bipolar symptoms, suicidal or homicidal ideations, and intent to harm himself. He complained of multiple symptoms after being struck by another patient, including a newfound stutter. This led to an emergency department visit, where he was diagnosed with a concussion. His stutter and other associated symptoms persisted until he admitted to fabricating them to avoid returning to juvenile detention. This case illustrates malingering for gain, specifically to evade legal consequences.

Malingering is the conscious and deliberate faking or exaggeration of illness or pain with the aim of attaining external benefits. These rewards encompass a variety of external gains, such as acquiring medication, avoiding legal consequences, and avoiding unfavorable work duties and expectations [4]. Identifying malingering in a healthcare setting is crucial due to its tremendous financial impact on healthcare expenditures. Some estimates indicate that malingering can result in costs exceeding $150 billion annually for the United States [5].

Our patient’s experience is interesting due to two separate instances where his malingering proved relevant to his medical care. His first episode began prior to his arrival at the psychiatric facility, where he was admitted for attempting suicide. Upon the initial psychiatric evaluation, the patient openly stated that he had created a noose but “knew it wouldn’t work” because it would not hold his body weight. He then proceeded to reenact his thought process without any prompting from the medical team, explaining how he specifically did this to avoid juvenile detention.

Although aware of his prior malingering behavior, concerns persisted regarding a possible brain injury following the assault. Further medical evaluation, including a CT head scan, was conducted to rule out traumatic brain injuries. However, it yielded no acute findings. Despite this, the patient's ongoing stutter and proposed vision changes raised suspicions. The differential diagnosis included concussion, conversion disorder, and malingering. Over subsequent days, the inconsistency of his stutter during interviews prompted him to confess to fabricating symptoms, prolonging his avoidance of juvenile detention.

Malingering is a diagnosis of exclusion. Therefore, it is necessary to prioritize the evaluation of other, more threatening differentials [6]. During patient evaluation following the altercation, the medical team considered the possibility that malingering might have been a motivating factor, particularly given the patient's history of such behavior upon admission to the psychiatric facility days earlier. However, with the patient having a head injury due to the altercation, malingering was not the primary focus for diagnosis.

According to the National Institutes of Health (NIH), no medicine or intervention can cure malingerers. Upon a detailed history, a malingerer may exhaust their excuses and give up. This is precisely what happened in this case, and we believe no intervention would have cured his malingering, as avoiding legal consequences was his motivation. A thorough neurological examination is necessary after any head injury, and imaging may certainly be warranted if any concerning symptoms are present. A case like this in which an assault was witnessed, despite a previous diagnosis of malingering, would likely order similar imaging to rule out serious injury.

Our patient admitted to faking the stutter and blurred vision after he was interviewed repeatedly over 24 hours. After several long conversations, his stutter became inconsistent, and that is what led the patient to confess. A detailed history pointed towards the more subjective neurologic symptoms as well as a thorough examination should be administered to identify any inconsistencies.

Malingering has no specific etiology, and determining its prevalence has been notoriously difficult. As mentioned earlier, children and adolescents are capable of deceptive behavior [7]. However, malingering is difficult to diagnose in children, requiring an increased level of suspicion. This could create the impression that malingering is rarer in children than it actually is. In light of these challenges, accurate recognition and diagnosis of malingering in children and adolescents demand heightened vigilance and a nuanced understanding of these deceptive behaviors to ensure appropriate intervention and support.

## Conclusions

This case report on a 15-year-old male discusses the complexity and diagnostic challenges of malingering in a healthcare setting. Malingering is the deliberate fabrication or exaggeration of symptoms for secondary gain. It poses a significant burden on the healthcare system and its resources, requiring an awareness of heightened suspicion and accurate identification. This case shows how malingering can manifest in adolescents, exemplifying the importance of a thorough assessment and the exclusion of other potential diagnoses. Early recognition of malingering will allow for better resource allocation and ensure that patients with true medical needs receive appropriate care. Healthcare professionals must remain aware and work towards applying a comprehensive approach by remaining vigilant for possible malingering and facilitating timely intervention and support for their patients.
